# Frontal-subcortical dysfunction in toxic oil syndrome: a proof-of-concept eye-tracking and cognitive study four decades after exposure

**DOI:** 10.3389/fnins.2025.1666809

**Published:** 2025-09-17

**Authors:** Julián Benito-León, José Lapeña-Motilva, Mariano Ruiz-Ortiz, Glen M. Doniger, Sonia Álvarez-Sesmero, Verónica Giménez de Béjar, María Antonia Nogales, Montserrat Morales, Ritwick Mondal, Shramana Deb, Fernando Bartolomé, Carolina Alquézar, Cecilia García-Cena

**Affiliations:** ^1^Department of Neurology, 12 de Octubre University Hospital, Madrid, Spain; ^2^Group of Neurodegenerative Diseases, Hospital Universitario 12 de Octubre Research Institute (imas12), Madrid, Spain; ^3^Network Center for Biomedical Research in Neurodegenerative Diseases (CIBERNED), Madrid, Spain; ^4^Department of Medicine, Complutense University, Madrid, Spain; ^5^Department of Clinical Research, NeuroTrax Corporation, Modiin, Israel; ^6^Department of Psychiatry, 12 de Octubre University Hospital, Madrid, Spain; ^7^Department of Internal Medicine, 12 de Octubre University Hospital, Madrid, Spain; ^8^Department of Neurology, Manipal Hospitals, Kolkata, India; ^9^Centre for Neurovascular Research, Manipal Hospitals, Kolkata, India; ^10^ETSIDI-Center for Automation and Robotics, Universidad Politécnica de Madrid, Madrid, Spain

**Keywords:** toxic oil syndrome, frontal-subcortical dysfunction, saccadic eye movements, cognitive impairment, eye-tracker

## Abstract

**Introduction:**

Toxic Oil Syndrome (TOS) emerged in Spain in 1981 after ingestion of rapeseed oil adulterated with aniline derivatives. More than four decades later, survivors continue to report cognitive complaints, but objective evidence of long-term dysfunction remains limited.

**Methods:**

In this case-control study, 47 TOS survivors and 44 matched healthy controls completed validated eye-tracking paradigms (visually guided, memory-guided, and antisaccade tasks) and a standardized neuropsychological battery. Groups did not differ significantly in age, sex, or education.

**Results:**

TOS survivors showed preserved performance on visually guided and memory-guided saccades, with no group differences in latency, gain, peak velocity, or spatial error (all *p* > 0.05). In contrast, they exhibited fewer correct antisaccades (mean 3.6 vs. 5.0; *p* = 0.029), more reflexive saccades (mean 7.0 vs. 5.7; *p* = 0.033), and increased backward reflexive saccades (mean 6.3 vs. 5.1; *p* = 0.040). Cognitive testing revealed selective impairments in executive function, attention, and processing speed, with preserved memory. Structural equation modeling confirmed that antisaccade impairment remained significant after adjusting for confounders and demonstrated an independent contribution of attention to correct antisaccade performance.

**Conclusion:**

Findings indicate persistent frontal-subcortical circuit dysfunction in TOS survivors, consistent with immune- or vascular-mediated injury patterns rather than progressive neurodegeneration. Eye-tracking provides a noninvasive biomarker of latent executive dysfunction and may be useful for long-term monitoring of populations exposed to environmental toxins.

## Introduction

Toxic oil syndrome (TOS) is a chronic, multisystem disease that emerged in Spain in May 1981 following the ingestion of rapeseed oil adulterated with aniline derivatives and illegally distributed for human consumption. The outbreak rapidly escalated, affecting over 20,000 individuals and causing more than 300 deaths within the first year (Abaitua Borda et al., 1998; Sánchez-Porro Valadés et al., 2003; Polentinos-Castro et al., 2021). The acute phase was characterized by eosinophilia, fever, myalgia, and pulmonary edema, later progressing to a chronic condition marked by scleroderma-like skin changes, peripheral neuropathy, muscular atrophy, and both pulmonary and neuropsychiatric manifestations (Gelpí et al., 2002; De La Paz et al., 2001). Although extensive multidisciplinary investigations were conducted, the specific toxic agent was never definitively identified. Nonetheless, fatty acid anilides and other byproducts of industrial oil denaturation were implicated based on chemical and toxicological analyses (Gelpí et al., 2002).

Kaufman and Krupp (1995) proposed that TOS and eosinophilia-myalgia syndrome may be part of a spectrum of chemically induced immune-mediated disorders involving the central nervous system. Their hypothesis initiated early discussions about mechanisms such as neuroinflammation and excitotoxicity. Notably, eosinophilia-myalgia syndrome itself is linked to neurocognitive dysfunction (Armstrong et al., 1997).

One of the earliest studies of cognitive impairment in TOS was reported in a small neuropsychological study by Del Ser et al. (1986), who found deficits in episodic and semantic memory within a few years of the onset. This was followed by a 12-year follow-up study by Kaufman et al. (1995), which offered clinical evidence of chronic morbidity in TOS survivors. Among 91 individuals re-evaluated more than a decade after exposure, over half reported persistent symptoms—fatigue, muscle cramps, arthralgias, subjective cognitive difficulties, and psychiatric complaints—despite being otherwise medically stable and lacking signs of a progressive neuromuscular disorder (Kaufman et al., 1995).

Further objective evidence came from a landmark case-referent study by De La Paz et al. (2003), conducted 18 years after the outbreak. TOS patients—particularly middle-aged women—performed significantly worse than matched controls on tests of motor strength, sensory function, and several neurocognitive domains such as memory, attention, processing speed, and psychomotor performance. The pattern of deficits indicated both peripheral and central nervous system involvement, with notable disruption of frontal-subcortical circuits (De La Paz et al., 2003).

Despite earlier findings, the long-term cognitive effects of TOS remain poorly understood. A recent 43-year follow-up case–control study found subtle yet measurable deficits in executive function, attention, and processing speed among TOS survivors, assessed with modern computerized tools (Lapeña-Motilva et al., 2025). In a separate biomarker study within the same group, slightly elevated serum neurofilament light chain levels were observed in TOS patients—mainly due to a few outliers—while levels of glial fibrillary acidic protein and phosphorylated tau 217 remained within normal ranges (Ruiz-Ortiz et al., 2025). These results suggest ongoing or residual neuronal injury in some individuals, without a biomarker pattern typical of progressive neurodegenerative disease (Ruiz-Ortiz et al., 2025).

Eye tracking provides an objective, noninvasive, high-resolution readout of cognitive control, as voluntary saccades are generated by frontal-subcortical circuits that subserve attention, executive control, and working memory. In particular, the frontal eye fields, dorsolateral prefrontal cortex, and anterior cingulate interact with basal ganglia loops and the superior colliculus to initiate or suppress gaze shifts; consequently, the antisaccade task—which requires suppression of a reflexive glance and generation of a willful movement in the opposite direction—is a sensitive probe of inhibitory control and working memory maintenance (Guerrero-Molina et al., 2021; García Cena et al., 2022a; García Cena et al., 2022b; Wolf et al., 2023; Benito-León et al., 2024; Leng et al., 2024; Benito-León et al., 2025). Across diverse neurological conditions, increased antisaccade error rates and prolonged latencies track frontal dysfunction, underscoring the utility of eye-movement metrics (e.g., error rates, reaction times) as surrogate markers of executive integrity (Guerrero-Molina et al., 2021; García Cena et al., 2022a; García Cena et al., 2022b; Wolf et al., 2023; Benito-León et al., 2024; Leng et al., 2024b; Benito-León et al., 2025).

To date, no studies have used eye tracking to examine cognitive dysfunction in TOS. We therefore employed three validated paradigms—visually guided saccades (basic sensorimotor function), memory-guided saccades (spatial working memory), and antisaccades (inhibitory control, executive function, and attention) to detect subtle deficits that may elude conventional testing (Guerrero-Molina et al., 2021; García Cena et al., 2022a; García Cena et al., 2022b; Wolf et al., 2023; Benito-León et al., 2024; Leng et al., 2024b; Benito-León et al., 2025). Given the chronic symptomatology of TOS, the predominance of dysexecutive features, and the absence of biomarker evidence for ongoing neurodegeneration, we hypothesized that TOS survivors would exhibit persistent oculomotor abnormalities, particularly in antisaccade performance, reflecting a long-standing disruption of frontal-subcortical circuits.

## Methods

### Population and recruitment procedures

Between April and June 2024, participants were recruited from the Madrid region, one of the most severely affected areas by the 1981 TOS outbreak. A case-control design was used: individuals with a confirmed diagnosis of TOS formed the exposed group, while healthy controls from the same geographic area served as the unexposed group. All assessments, including interviews and cognitive testing, were conducted at the 12 de Octubre University Hospital in Madrid.

### Participant selection

TOS cases were identified based on diagnostic criteria established in earlier research (Lapeña-Motilva et al., 2025; Ruiz-Ortiz et al., 2025). Eligible individuals had experienced either the acute or chronic phase of the illness. The acute phase was characterized by alveolar–interstitial infiltrates and/or pleural effusion, along with absolute eosinophilia of more than 500 cells/mm^3^. The chronic phase criteria included persistent myalgia with eosinophilia and/or at least one of the following: scleroderma-like skin changes, peripheral neuropathy, pulmonary hypertension, or liver involvement.

Patients were enrolled through the 12 de Octubre University Hospital’s dedicated TOS unit—the only center in Spain specialized in the long-term follow-up of affected individuals. Recruitment was consecutive until 50 TOS cases were included. The control group consisted of 50 healthy individuals from the same community, selected from acquaintances or friends of the patients. Controls were matched by age (±5 years), sex, and educational level to ensure comparability between groups. Given the fixed size of the eligible clinic cohort and the absence of prior eye-tracking effect-size estimates in TOS, we prospectively set a pragmatic target of 50 cases and 50 matched controls based on feasibility within the recruitment window, acknowledging that a formal *a priori* power calculation was not possible in this rare disease context.

Exclusion criteria for both groups included any history of neurodegenerative disease (e.g., Alzheimer’s or Parkinson’s disease), stroke, chronic kidney disease, alcohol abuse, or prior traumatic injury involving the central or peripheral nervous system. Identical criteria were applied to controls to maintain methodological rigor.

### Assessments and measures

#### Demographic and clinical characteristics

Participant data—including age, sex, educational background, medical history, and current medications—were obtained through a structured clinical interview and after revision of their medical records. Educational attainment was recorded in four categories: illiterate, primary, secondary and higher studies.

#### Fatigue evaluation

Fatigue was measured using the Fatigue Impact Scale for Daily Use (D-FIS; Martínez-Martín et al., 2006; Benito-León et al., 2007), a validated 8-item self-report tool designed to assess how fatigue interferes with daily activities. Each item is scored on a five-point Likert scale, from 0 (no problem) to 4 (extreme problem), yielding a total score that reflects the extent of fatigue-related impact on functioning.

#### Health-related quality of life

The EuroQol instrument was used to assess perceived health-related quality of life (Badia et al., 2001). This tool includes two parts: the EQ-5D descriptive system and a visual analog scale (EQ VAS). The EQ-5D has five domains—mobility, self-care, usual activities, pain/discomfort, and anxiety/depression—each rated on a three-level scale (no problems, moderate problems, severe problems; Badia et al., 2001). Responses create a health profile, from which an index score is calculated using standardized European value sets (Badia et al., 2001). This score ranges from 1, indicating perfect health, to 0, representing death, and negative scores indicate states worse than death (Badia et al., 2001). The EQ VAS enables participants to rate their overall health on a vertical scale ranging from 0 (the worst imaginable health) to 100 (the best imaginable health; Badia et al., 2001).

#### Assessment of depressive symptoms

Depression severity was assessed using the Beck Depression Inventory-II (BDI-II) (Beck et al., 1996), a 21-item self-report questionnaire widely used in clinical and research settings (Beck et al., 1996). Each item reflects a symptom commonly associated with depression and is scored from 0 (absent) to 3 (severe), based on experiences over the prior 2 weeks (Beck et al., 1996). Higher total scores indicate more severe depressive symptoms (Beck et al., 1996).

#### Assessment of anxiety symptoms

Anxiety was measured using the Beck Anxiety Inventory (BAI; Beck et al., 1988), a validated instrument consisting of 21 items evaluating common symptoms of anxiety. Participants rate how much they have been bothered by each symptom in the past week using a 4-point scale, ranging from 0 (not at all) to 3 (severely bothered). Total scores range from 0 to 63, with higher scores indicating greater severity of anxiety (Beck et al., 1988).

#### Cognitive function testing

Cognitive performance was assessed using the NeuroTrax computerized system, which offers standardized and validated tests across multiple cognitive areas (Doniger et al., 2005; Schweiger et al., 2007; Dwolatzky et al., 2003). Due to limited time, the assessment focused on specific domains: verbal and non-verbal memory, attention (measured through Go-NoGo and Stroop Interference tasks), processing speed, and executive function (evaluated via the Go-NoGo, Stroop Interference, and Catch Game tasks; Doniger et al., 2005; Schweiger et al., 2007; Dwolatzky et al., 2003). All testing was conducted in Spanish, the native language of all participants.

#### Eye movement assessments

**Figure 1:**
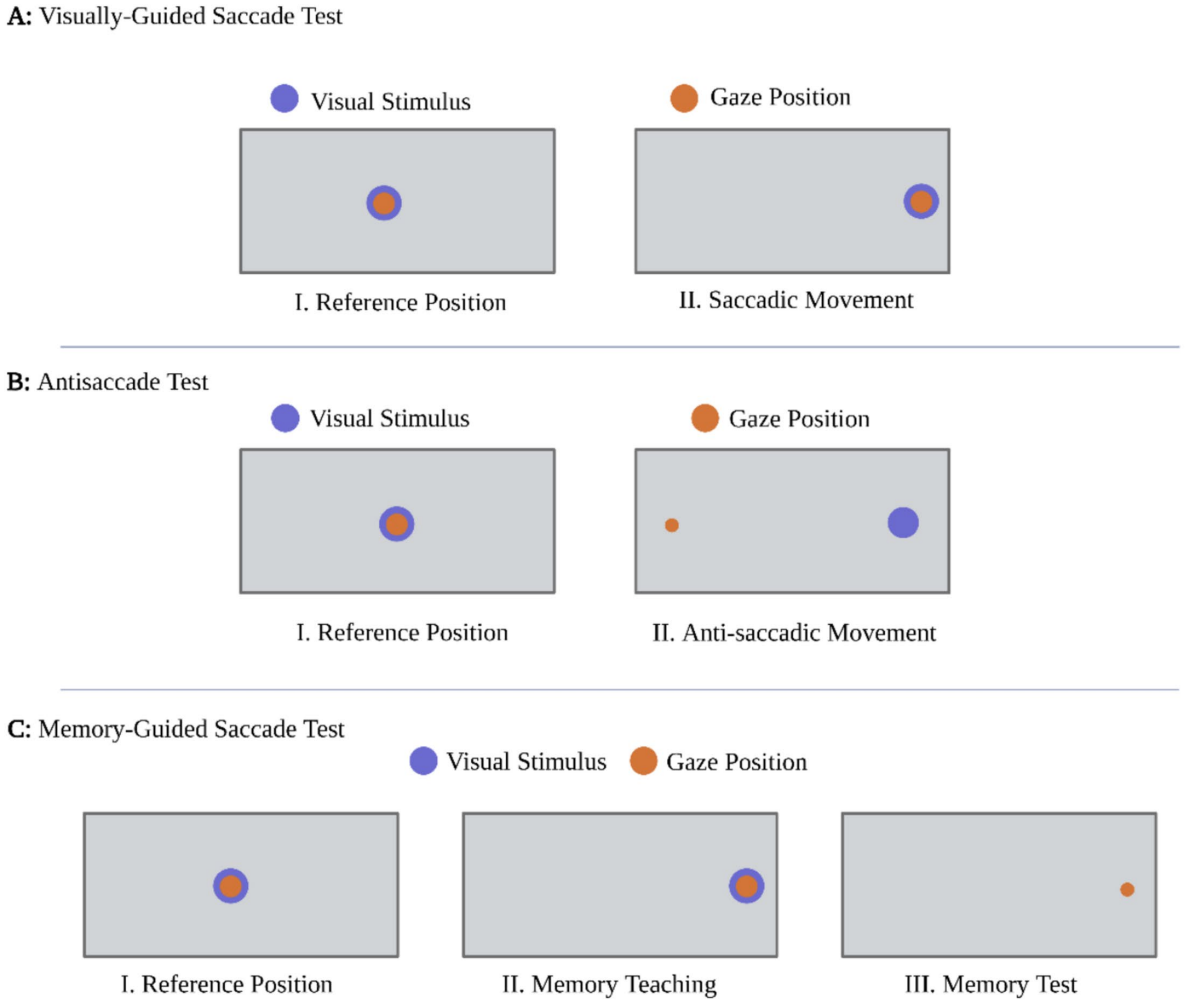
Schematic of eye-tracking paradigms. **(A)** Visually guided saccades. Participants fixated a central target (I) and then made a saccade to a peripheral stimulus (II). This task indexes basic sensorimotor oculomotor function (latency, gain, peak velocity). **(B)** Antisaccades. Participants fixated centrally (I) and, when a peripheral stimulus appeared, suppressed the automatic response toward it and instead generated a saccade to the mirror-symmetric location on the opposite side (II). This task indexes inhibitory control, executive function, and attention. **(C)** Memory-guided saccades. Participants fixated centrally (I); a peripheral cue briefly appeared to be remembered (II); after a short delay, they made a saccade to the remembered location (III). This task probes spatial working memory and delayed oculomotor planning. Blue dot = visual stimulus; orange dot = gaze position. Each task comprised 22 trials.

All eye-tracking evaluations were conducted in a dimly lit room, with recordings made monocularly from the dominant eye of each participant. Standardized instructions were provided, and a practice trial ensured proper understanding of the task. Saccades with latencies below 80 milliseconds and those occurring during blinks were excluded from analysis. The protocol included three well-established paradigms: visually guided saccades, memory-guided saccades, and the antisaccade task, all of which were performed on a horizontal axis (Figure 1) (Guerrero-Molina et al., 2021; García Cena et al., 2022a; García Cena et al., 2022b; Benito-León et al., 2024; Benito-León et al., 2025). We followed our published eye-tracking protocol to ensure reproducibility and enable direct comparison with earlier measurements (García Cena et al., 2020). A brief antisaccade paradigm (22 trials) was implemented to reduce fatigue and maintain calibration stability, consistent with clinical and instrument validation protocols (Hellmuth et al., 2012; Brooks et al., 2019).

- Visually guided saccades

With central fixation maintained, a peripheral target appeared at ±5°, ±10°, or ±20° along the horizontal meridian in pseudorandom order; participants were instructed to look at the target as quickly and accurately as possible and then return their gaze to the central fixation. A correct visually guided saccade was defined as the first goal-directed saccade to the target location; a backward saccade was the subsequent return movement to fixation.Measures included: number of correct saccades; number of errors; latency (ms) from stimulus onset to saccade onset; peak velocity (°/s); and gain (executed amplitude divided by target amplitude). Spatial error relative to the target was assessed separately for hypermetria (overshoot, positive degrees of visual angle) and hypometria (undershoot, negative degrees); we also counted the number of hypermetric saccades and the number of hypometric saccades. For the return movement, we recorded the number of correct backward saccades; number of backward errors; backward latency (ms); and peak backward velocity (°/s).

- Memory-guided saccades

While maintaining central fixation, a peripheral cue briefly appeared and then disappeared; after a short delay, participants made a saccade to the remembered location; then returned their gaze to the center once the fixation point reappeared. A correct memory-guided saccade was the first goal-directed saccade toward the remembered location; errors were first saccades directed elsewhere.Gain was calculated as executed amplitude divided by target amplitude; signed spatial error was the landing-position error (degrees of visual angle) relative to the cued location (positive = overshoot/hypermetria; negative = undershoot/hypometria).Measures included: number of correct memory-guided saccades; number of errors; latency; peak velocity; gain; hypermetric and hypometric spatial errors; number of correct backward memory-guided saccades (return movements to center); number of backward errors; backward latency; and peak backward velocity.

- Antisaccade task

Participants maintained central fixation; when a peripheral stimulus appeared, they were instructed to suppress the automatic glance toward it and instead generate a saccade to the mirror-symmetric location on the opposite side. A correct antisaccade was the first goal-directed saccade from fixation (screen center) to the mirror-symmetric location; a reflexive saccade was an initial saccade toward the cue, followed by a corrective movement to the mirror position; any other eye movement was considered an antisaccade error.Backward eye movements—whether backward antisaccades or backward reflexive saccades—were defined as saccades from the mirror-symmetric location back to the screen center.Measures included: number of correct antisaccades; number of antisaccade errors; antisaccade latency (ms); number of correct backward antisaccades; number of backward antisaccade errors; backward antisaccade latency (ms); number of reflexive saccades; reflexive saccade latency (ms); number of backward reflexive saccades; backward reflexive saccade latency (ms); and reflexive saccade duration (ms).

### Standard protocol approvals, registrations, and patient consents

This study was approved by the Institutional Review Board (IRB) at the 12 de Octubre University Hospital in Madrid, Spain (CEIC codes: 17/035 and 23/616). It was conducted in accordance with the Declaration of Helsinki. Written (signed) informed consent was obtained from all participants.

### Statistical analyses

Trial-level metrics were aggregated for each participant (means for normally distributed continuous variables, medians for skewed distributions, and totals for counts) and analyzed at the group level. All statistical analyses and figure creation were performed using Python (v3.12.2). The Python packages used included pandas (v2.2.3) for data management, TableOne (v0.9.1) for descriptive statistics, and semopy (v2.3.11) for structural equation modeling (SEM). Continuous variables were tested for normality with the Kolmogorov–Smirnov test. Between-group comparisons of demographic, cognitive, and eye-tracking data were made using independent-sample t-tests for normally distributed variables and Mann–Whitney U tests when normality assumptions were not met. Chi-square tests were used to assess group differences in categorical variables.

In this study, cognitive performance data obtained from the NeuroTrax computerized assessment were standardized by transforming raw scores into z-scores using the control group’s mean and standard deviation as reference. Z-scores were computed for all participants, regardless of group assignment. Consistent with previously published NeuroTrax-based protocols (Lapeña-Motilva et al., 2025; Doniger et al., 2005; Schweiger et al., 2007), domain-level indices were derived by averaging normalized values from domain-related test scores (e.g., accuracy, response time). A global cognitive score was then calculated by averaging across all domain indices.

We developed multivariate models to evaluate the influence of clinical, psychological, and fatigue-related variables on eye-tracking measures that were significant in univariate analyses. After adjusting for age, sex, and education (Fjell et al., 2010; Roe et al., 2007; Seblova et al., 2020), we entered clinical comorbidities with established associations to cognitive decline—arterial hypertension and diabetes mellitus—because both were more prevalent in the TOS group and have recognized links to cognitive impairment (Bermejo-Pareja et al., 2010; Shome et al., 2025). In contrast, polyneuropathy was not modeled as a confounder because it is an intrinsic feature of TOS and has not been shown to contribute to cognitive dysfunction. Psychological symptoms (BDI-II and BAI) and fatigue (D-FIS) were then included as predictors. To quantify both direct and indirect pathways, we used SEM. Because BDI-II and BAI scores were highly collinear, we derived a single mood-distress composite via principal component analysis; the first component explained 90.92% of the shared variance and was carried forward as the Composite Variable for Depression and Anxiety (CVDA) in all multivariable and SEM models.

All *p*-values were two-tailed, and statistical significance was defined as *p* < 0.05. Given the proof-of-concept and exploratory nature of this study, no corrections for multiple comparisons were applied. This decision is supported by established recommendations that highlight the potential for such adjustments to increase Type II error unnecessarily and emphasize that they are not essential in hypothesis-generating research (Rothman, 1990; Perneger, 1998; Bender and Lange, 2001).

## Results

Of the 50 TOS patients and 50 healthy controls initially recruited, valid eye-tracking data were obtained for 47 and 44 participants, respectively. Participants were excluded due to acquisition-related issues (e.g., inadequate task compliance, calibration errors) or poor preprocessing quality (signal loss, artifacts, unstable tracking) that prevented analysis. As this was an exploratory, proof-of-concept study in a rare condition, a formal *a priori* sample-size calculation was not feasible. After data collection, a *post-hoc* power analysis (two-tailed independent-samples t-test, *α* = 0.05) showed that the analytic sample (*N* = 91) had approximately 83.8% power to detect the observed medium effect size (Cohen’s d ≈ 0.62) for the global cognitive score. Accordingly, the study was not powered to detect small between-group differences.

Demographic and neuropsychological data are summarized in Table 1. Groups were comparable in terms of age (mean age ~59 years), sex distribution (predominantly female), and educational attainment (*p* > 0.05 for all comparisons). However, TOS patients exhibited significantly higher prevalence of diabetes mellitus (25.5% vs. 2.3%), arterial hypertension (66.0% vs. 20.5%), and polyneuropathy (72.3% vs. 0%; all *p* < 0.005). TOS patients also showed markedly worse scores in health-related quality of life (EuroQol-5D index), fatigue (D-FIS), anxiety (BAI), and depression (BDI-II; all *p* < 0.001). Cognitively, they demonstrated significantly lower global cognitive scores (*p* = 0.007), with prominent deficits in executive function (*p* = 0.017), attention (*p* = 0.014), and information processing speed (*p* = 0.009). No significant differences were observed in memory subdomains.

**Table 1:** Demographic and clinical characteristics of the study population by group.

Variable	Controls (*N* = 44)	Patients (*N* = 47)	*p* value
Age, mean (standard deviation)	58.3 (8.1)	60.1 (8.0)	0.286^a^
Female, %	28 (63.6)	35 (74.5)	0.373^b^
Education, N (%)			0.226^b^
Illiterate	1 (2.3)	3 (6.4)	
Primary studies	16 (36.4)	18 (38.3)	
Secondary studies	12 (27.3)	18 (38.3)	
Higher studies	15 (34.1)	8 (17.0)	
Diabetes mellitus, N (%)	1 (2.3)	12 (25.5)	**0.004** ^**b**^
Arterial hypertension, N (%)	9 (20.5)	31 (66.0)	**<0.001** ^**b**^
Polyneuropathy, N (%)	0 (0.0)	34 (72.3)	**<0.001** ^**b**^
EuroQol 5-Dimensions index, median [Q1, Q3]	0.9 [0.7, 1.0]	0.4 [0.1, 0.8]	**<0.001** ^**c**^
Fatigue Impact Scale for Daily Use, mean (standard deviation)	4.9 (6.3)	19.2 (8.5)	**<0.001** ^**a**^
Beck Anxiety Inventory, median [Q1, Q3]	4.0 [1.8, 11.8]	22.0 [14.0, 30.5]	**<0.001** ^**c**^
Beck Depression Inventory, median [Q1, Q3]	5.0 [2.0, 14.0]	19.0 [11.5, 28.5]	**<0.001** ^**c**^
Global Cognitive Score, mean (standard deviation)^d^	0.0 (0.7)	−0.5 (0.9)	**0.007** ^**a**^
Cognitive domains^d^			
Memory, mean (standard deviation)	0.0 (0.9)	−0.3 (0.9)	0.209^a^
Immediate memory, mean (standard deviation)	0.0 (0.9)	−0.3 (0.9)	0.257^a^
Delayed memory, mean (standard deviation)	0.0 (0.9)	−0.3 (1.0)	0.149^a^
Executive function, mean (standard deviation)	0.0 (0.8)	−0.4 (1.0)	**0.017** ^**a**^
Attention, median [Q1, Q3]	0.1 [−0.2,0.5]	−0.2 [−1.4,0.3]	**0.014** ^**c**^
Information processing speed, mean (standard deviation)	0.0 (0.8)	−0.6 (1.0)	**0.009** ^**a**^

a Student t test.

b Chi-square test or Fisher’s exact test, as appropriate.

c Mann–Whitney U test.^d^ Z-scores (control group as reference, see Methods). Bold values indicate statistical significance at *p* < 0.05.

### Oculomotor tasks

#### Visually guided saccades

**Figure 2:**
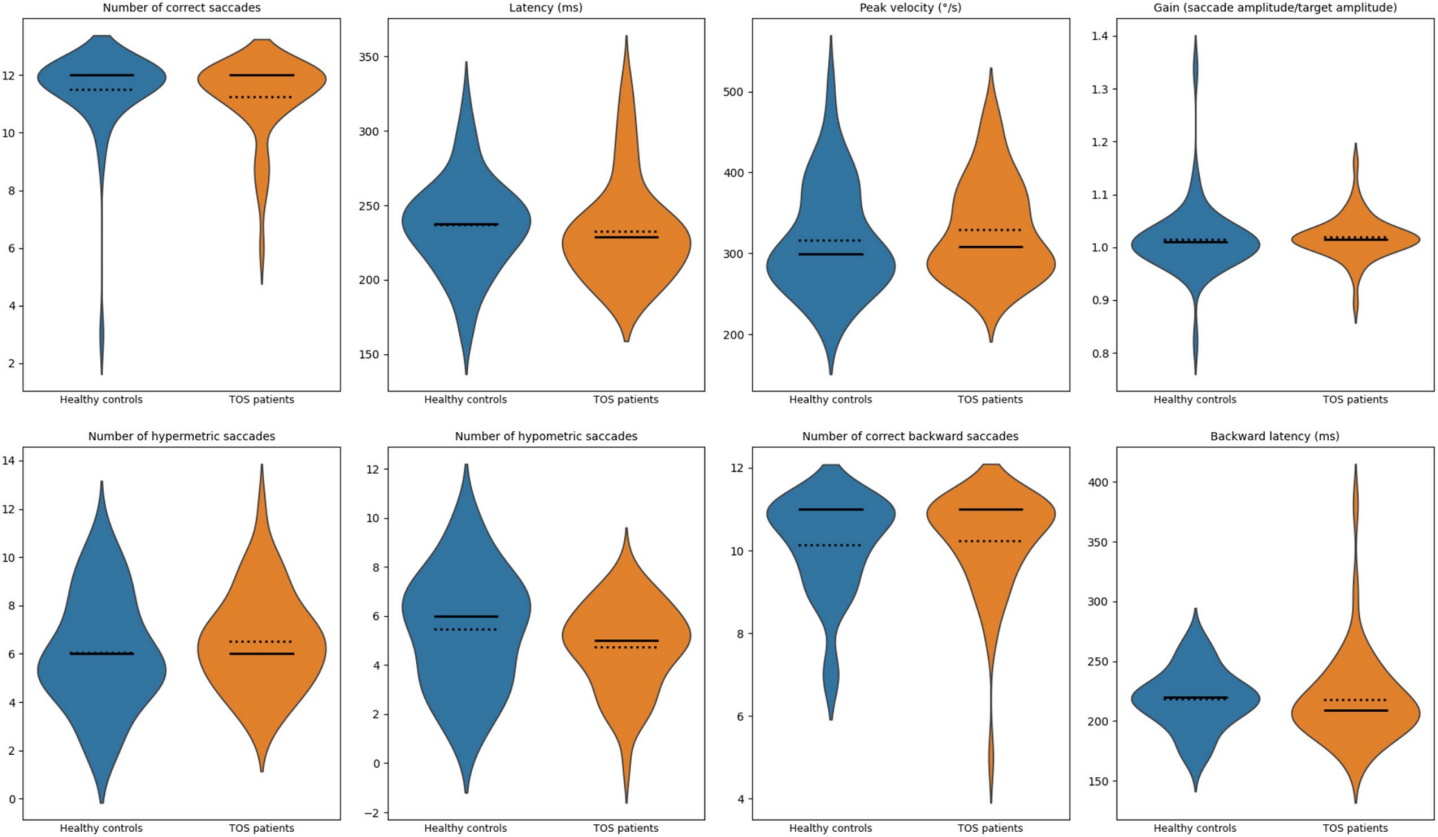
Violin plots of visually guided saccade measures. Violin plots show the distribution of oculomotor measures for healthy controls (blue) and patients with toxic oil syndrome (orange). From left to right, top row: number of correct saccades, latency (ms), peak velocity (°/s), and gain (executed amplitude divided by target amplitude). Bottom row: number of hypermetric saccades, number of hypometric saccades, number of correct backward saccades, and backward latency (ms). Solid lines indicate group medians; dotted lines indicate group means.

TOS survivors exhibited preserved performance across all visually guided saccade measures. Figure 2 shows the distribution of parameters recorded during the task: (1) number of correct saccades, (2) latency (ms), (3) peak velocity (°/s), (4) gain (executed amplitude divided by target amplitude), (5) number of hypermetric saccades, (6) number of hypometric saccades, (7) number of correct backward saccades, and (8) backward latency (ms). As detailed in Table 2, no statistically significant differences were observed between groups in any parameter (all *p* > 0.05). TOS participants showed subtle, non-significant trends toward slightly faster backward latencies and marginally higher peak velocities. Gain values remained tightly centered around 1.0 in both groups, indicating accurate amplitude scaling. Variability in spatial error (hypermetric and hypometric saccades) was also comparable. These findings indicate that visually guided saccades are preserved in TOS survivors, with no evidence of disrupted basic oculomotor function.

**Table 2:** Visually guided and memory-guided saccade and antisaccade tests.

	Controls (*N* = 44)	Patients (*N* = 47)	*p* value
Visually guided saccade test			
Number of correct saccades, median [Q1, Q3]	12.0 [12.0,12.0]	12.0 [11.0, 12.0]	0.149^a^
Number of errors, median [Q1, Q3]	0.0 [0.0, 0.0]	0.0 [0.0, 1.0]	0.149^a^
Latency (ms), mean (standard deviation)	236.8 (30.8)	232.8 (33.9)	0.562^b^
Peak velocity (°/s), mean (standard deviation)	316.7 (65.9)	329.6 (59.1)	0.333^b^
Gain (saccade amplitude/target amplitude), median [Q1, Q3]	1.0 [1.0, 1.0]	1.0 [1.0, 1.0]	0.096^a^
Spatial error relative to target – hypermetria (° visual angle), median [Q1, Q3]	0.4 [0.3, 0.6]	0.4 [0.3, 0.8]	0.831^a^
Number of hypermetric saccades, mean (standard deviation)	6.0 (2.3)	6.5 (2.0)	0.298^b^
Spatial error relative to target – hypometria (° visual angle), median [Q1, Q3]	−0.5 [−0.8, −0.3]	−0.5 [−0.8, −0.3]	0.548^a^
Number of hypometric saccades, mean (standard deviation)	5.5 (2.3)	4.7 (1.7)	0.105^b^
Number of correct backward saccades, median [Q1, Q3]	11.0 [9.8,11.0]	11.0 [10.0, 11.0]	0.615^a^
Number of backward errors, median [Q1, Q3]	0.0 [0.0, 1.2]	0.0 [0.0, 1.0]	0.615^a^
Backward latency (ms), median [Q1, Q3]	219.6 [205.4, 231.1]	209.2 [197.5, 230.2]	0.257^a^
Peak backward velocity (°/s), mean (standard deviation)	320.5 (70.9)	323.0 (58.2)	0.859^b^
Memory-guided saccade test			
Number of correct memory-guided saccades, mean (standard deviation)	8.3 (2.8)	7.6 (3.5)	0.286^b^
Number of errors, mean (standard deviation)	3.7 (2.8)	4.4 (3.5)	0.286^b^
Latency (ms), median [Q1, Q3]	413.5 [307.3, 561.6]	456.0 [355.6, 591.1]	0.331^a^
Peak velocity (°/s), mean (standard deviation)	285.5 (84.9)	265.8 (70.4)	0.244^b^
Gain (saccade amplitude/target amplitude), mean (standard deviation)	0.9 (0.3)	0.9 (0.3)	0.573^b^
Spatial error relative to cued location – hypermetria (° visual angle), median [Q1, Q3]	0.6 [0.3, 1.3]	1.0 [0.3, 1.8]	0.130^a^
Number of hypermetric saccades, mean (standard deviation)	5.2 (3.0)	5.3 (3.1)	0.915^b^
Spatial error relative to cued location – hypometria (° visual angle), median [Q1, Q3]	−3.1 [−5.1, −0.9]	−4.0 [−6.8, −1.1]	0.435^a^
Number of hypometric saccades, mean (standard deviation)	4.6 (3.1)	4.6 (2.7)	0.980^b^
Number of correct backward memory-guided saccades, median [Q1, Q3]	8.0 [5.0, 10.0]	7.0 [4.0, 10.0]	0.446^a^
Number of backward errors, median [Q1, Q3]	3.0 [1.0, 6.0]	4.0 [1.0, 7.0]	0.446^a^
Backward latency (ms), median [Q1, Q3]	332.6 [299.1, 398.3]	326.2 [270.4, 451.2]	0.990^a^
Peak backward velocity (°/s), mean (standard deviation)	286.8 (97.7)	288.2 (94.0)	0.948^b^
Antisaccade test			
Number of correct antisaccades, mean (standard deviation)	5.0 (3.1)	3.6 (3.0)	**0.029** ^**b**^
Number of antisaccade errors, median [Q1, Q3]	0.0 [0.0,1.5]	1.0 [0.0, 2.0]	0.418^a^
Antisaccade latency (ms), median [Q1, Q3]	370.7 [340.1,428.6]	383.0 [319.5, 449.4]	0.854^a^
Number of correct backward antisaccades, mean (standard deviation)	4.1 (2.5)	3.3 (2.8)	0.136^b^
Number of backward antisaccade errors, median [Q1, Q3]	1.0 [0.0,2.0]	1.0 [0.0, 2.0]	0.475^a^
Backward antisaccade latency (ms), median [Q1, Q3]	355.8 [312.4,417.7]	361.3 [316.0, 438.3]	0.723^a^
Number of reflexive saccades, mean (standard deviation)	5.7 (2.9)	7.0 (3.1)	**0.033** ^**b**^
Reflexive saccade latency (ms), median [Q1, Q3]	332.0 [302.5,354.9]	315.9 [287.4, 361.1]	0.248^a^
Number of backward reflexive saccades, mean (standard deviation)	5.1 (2.5)	6.3 (2.7)	**0.040** ^**b**^
Backward reflexive saccade latency (ms), mean (standard deviation)	436.2 (137.2)	445.8 (144.1)	0.745^b^
Reflexive saccade duration (ms), median [Q1, Q3]	270.4 [198.1,342.0]	307.3 [226.1, 388.2]	0.251^a^

a Mann-Whitney U test.

b Student t test.Bold values indicate statistical significance at *p* < 0.05.

#### Memory-guided saccades

**Figure 3:**
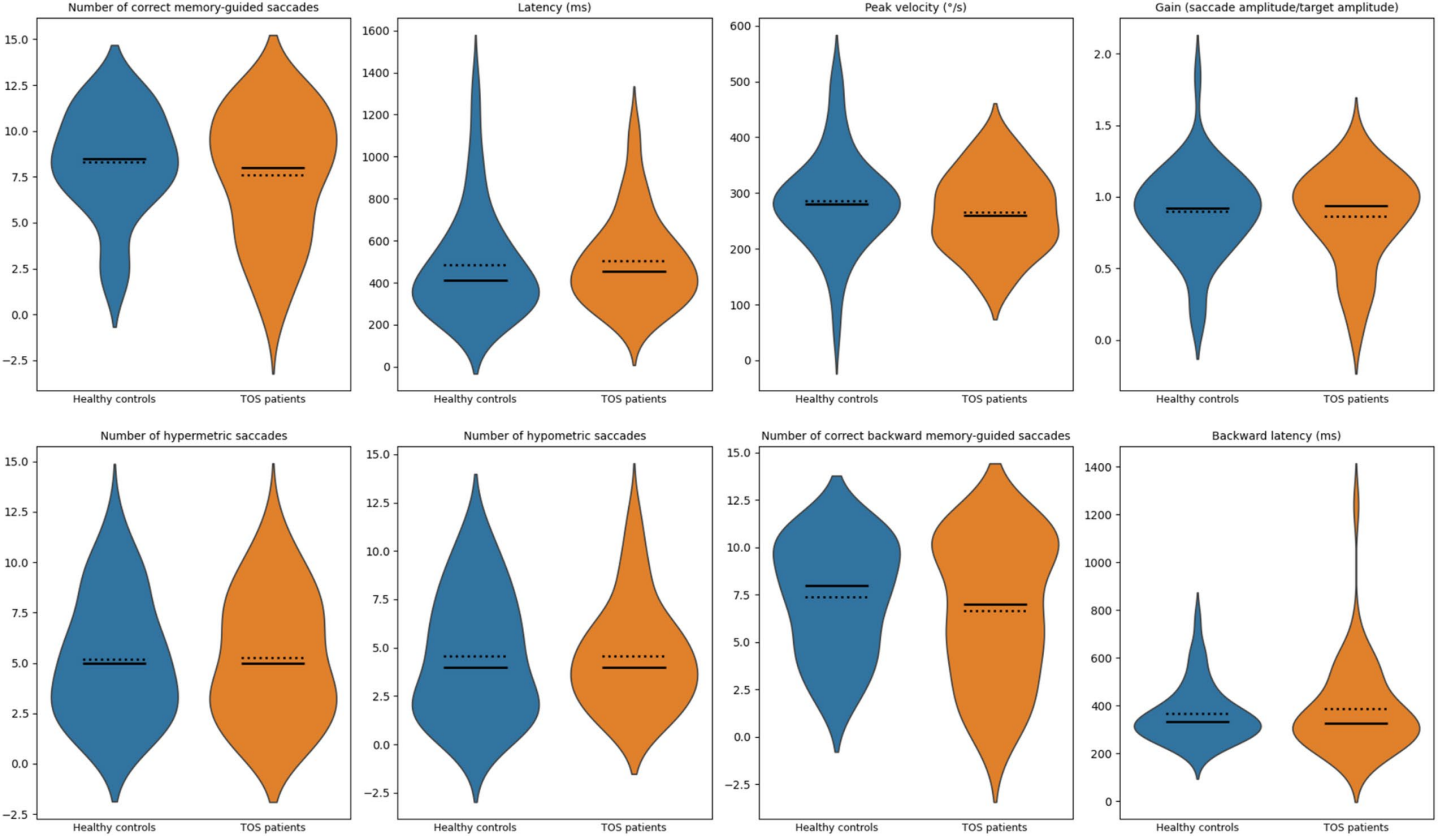
Violin plots of memory-guided saccade measures. Violin plots show the distribution of oculomotor measures for healthy controls (blue) and patients with toxic oil syndrome (orange). Top row (left to right): number of correct memory-guided saccades, latency (ms), peak velocity (°/s), and gain (executed amplitude divided by target amplitude). Bottom row (left to right): number of hypermetric saccades, number of hypometric saccades, number of correct backward memory-guided saccades, and backward latency (ms). Solid lines indicate group medians; dotted lines indicate group means.

Performance on the memory-guided saccade task was also preserved in TOS survivors. Figure 3 displays the distribution of measures: (1) number of correct memory-guided saccades, (2) latency (ms), (3) peak velocity (°/s), (4) gain, (5) number of hypermetric saccades, (6) number of hypometric saccades, (7) number of correct backward memory-guided saccades, and (8) backward latency (ms). Compared with controls, TOS participants exhibited mild, non-significant tendencies toward longer latencies, lower peak velocities, and greater dispersion of spatial error (including hypermetric and hypometric errors). Gain values remained close to 1.0 in both groups. As summarized in Table 2, no statistically significant group differences were found in any parameter (all *p* > 0.05). These results indicate that memory-guided saccadic performance remains largely intact in TOS, suggesting that the integration of spatial working memory with oculomotor planning is preserved.

#### Antisaccades

**Figure 4:**
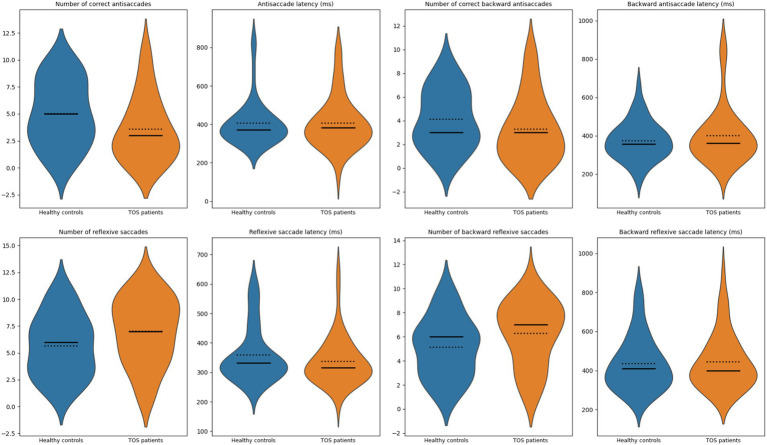
Violin plots of antisaccade measures. Violin plots illustrate the distribution of measures for healthy controls (blue) and patients with toxic oil syndrome (orange). Top row: number of correct antisaccades, antisaccade latency (ms), number of correct backward antisaccades, and backward antisaccade latency (ms). Bottom row: number of reflexive saccades, reflexive saccade latency (ms), number of backward reflexive saccades, and backward reflexive saccade latency (ms). Solid lines indicate group medians; dotted lines indicate group means.

In contrast, TOS survivors demonstrated impaired antisaccade performance accompanied by increased reflexive responses, consistent with deficits in attentional and executive control. Figure 4 illustrates the distribution of eight parameters: (1) number of correct antisaccades, (2) antisaccade latency (ms), (3) number of correct backward antisaccades, (4) backward antisaccade latency (ms), (5) number of reflexive saccades, (6) reflexive saccade latency (ms), (7) number of backward reflexive saccades, and (8) backward reflexive saccade latency (ms). Compared to matched controls, TOS patients exhibited significantly fewer correct antisaccades (mean 3.6 vs. 5.0; *p* = 0.029). In parallel, they showed more reflexive saccades (mean 7.0 vs. 5.7; *p* = 0.033) and a modest increase in backward reflexive saccades (mean 6.3 vs. 5.1; *p* = 0.040; Table 2). No significant differences were observed in antisaccade latency (*p* = 0.854), backward antisaccade latency (*p* = 0.723), or reflexive saccade duration (*p* = 0.251).

To further examine these associations, we constructed structural equation models (SEMs) adjusting for potential confounders. Attention was specified a priori as the sole mediator because antisaccade performance relies on top-down attentional control, and including multiple, correlated domains would over-parameterize the model in this sample (Munoz and Everling, 2004; Koçoğlu et al., 2021) We intentionally excluded formal executive function scores because of their strong conceptual and statistical overlap with antisaccade performance, which itself is a sensitive and well-established indicator of executive control (Nieuwenhuis et al., 2004; Mirsky et al., 2011). Similarly, we did not include the global cognitive score, which aggregates across multiple domains not directly related to antisaccade generation and could obscure specific associations with oculomotor measures.

**Figure 5:**
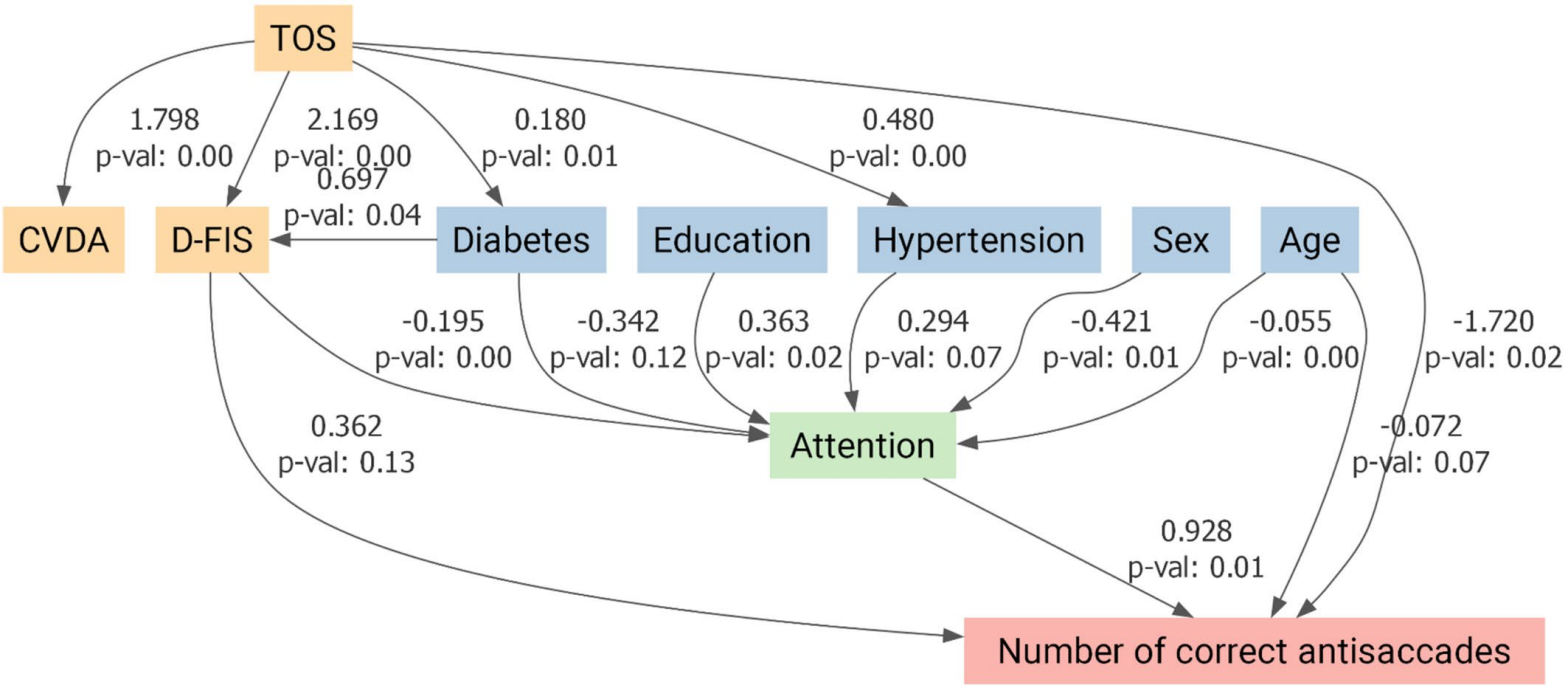
Structural equation model for antisaccade performance in toxic oil syndrome (TOS) survivors. Path diagram illustrating the direct and indirect effects of TOS on the number of correct antisaccades, adjusted for covariates. Predictor variables included a composite variable for depression and anxiety (CVDA), fatigue (D-FIS), diabetes mellitus, arterial hypertension, education, sex, and age. Attention was modeled as a potential mediator. Arrows represent standardized regression coefficients with associated *p*-values. TOS showed a significant direct negative effect on antisaccade performance (*β* = −1.720, *p* = 0.02). At the same time, attention was positively associated with performance (*β* = 0.928, *p* = 0.01), indicating that both disease status and attentional processes independently influenced antisaccade measures. CVDA, Composite Variable for Depression and Anxiety; D-FIS, Daily Fatigue Impact Scale.

**Figure 6:**
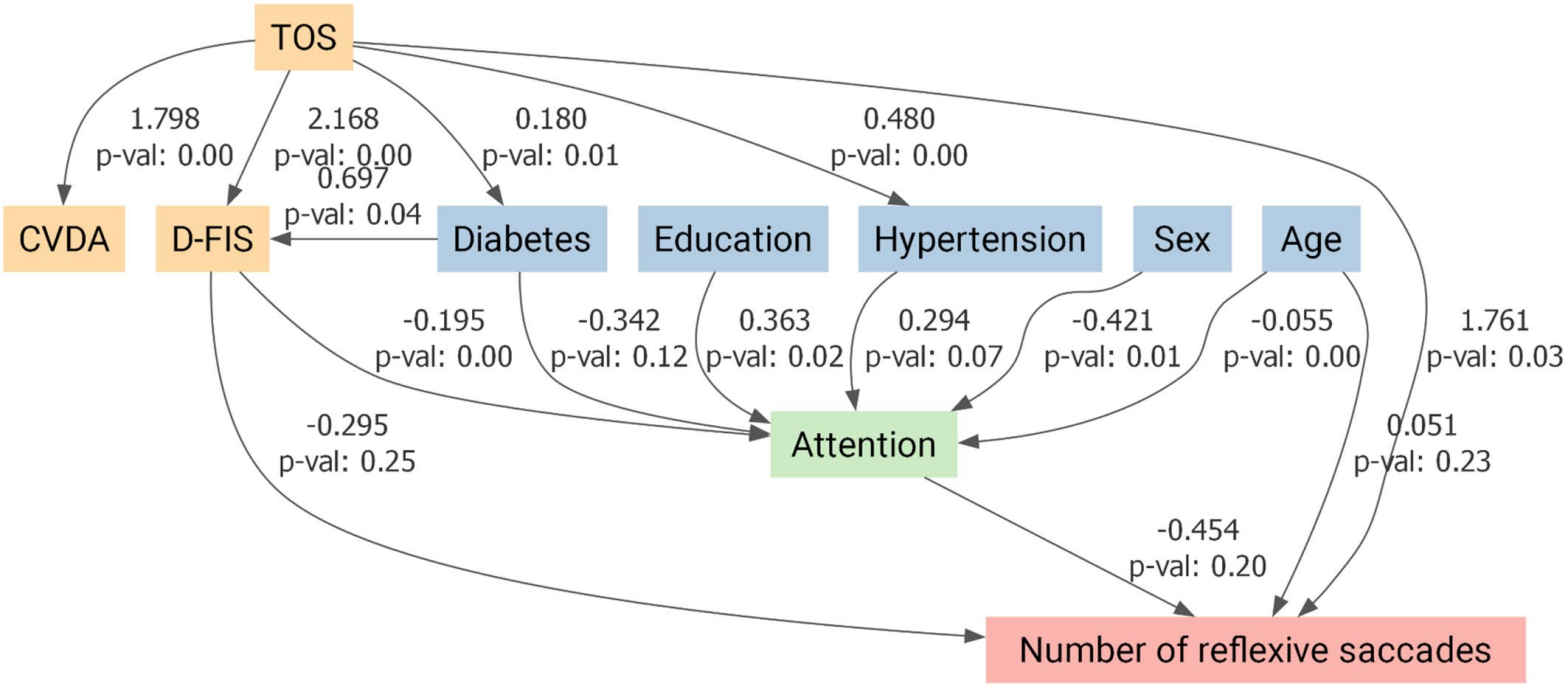
Structural equation model for reflexive saccade performance in toxic oil syndrome (TOS) survivors. Path diagram illustrating the direct and indirect effects of TOS on the number of reflexive saccades, adjusted for covariates. Predictor variables included a composite variable for depression and anxiety (CVDA), fatigue (D-FIS), diabetes mellitus, arterial hypertension, education, sex, and age. Attention was modeled as a potential mediator. Arrows represent standardized regression coefficients with associated *p*-values. TOS exerted a significant direct positive effect on reflexive saccade frequency (*β* = 1.761, *p* = 0.03), while attention showed no significant mediation effect. CVDA, Composite Variable for Depression and Anxiety; D-FIS, Daily Fatigue Impact Scale.

**Figure 7:**
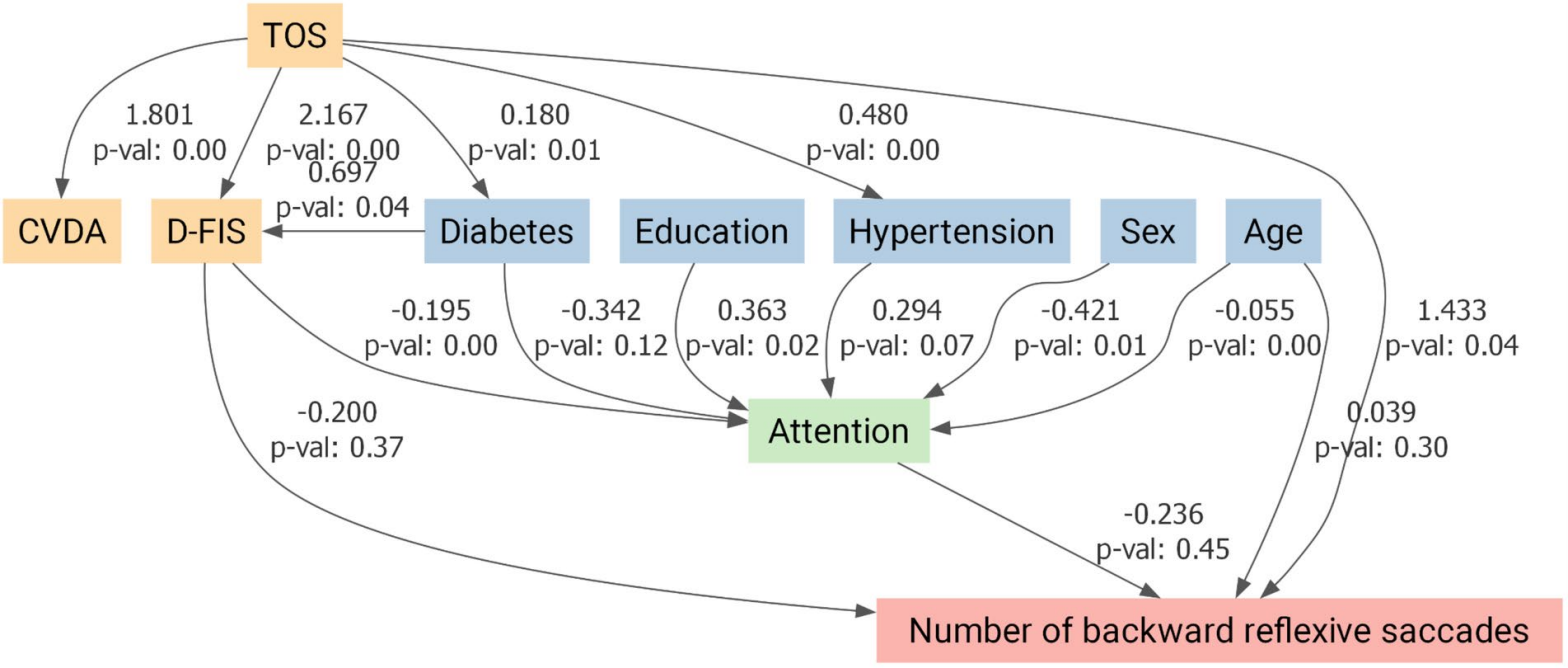
Structural equation model for backward reflexive saccade performance in toxic oil syndrome (TOS) survivors. Path diagram illustrating the direct and indirect effects of TOS on the number of backward reflexive saccades, adjusted for covariates. Predictor variables included a composite variable for depression and anxiety (CVDA), fatigue (D-FIS), diabetes mellitus, arterial hypertension, education, sex, and age. Attention was modeled as a potential mediator. Arrows represent standardized regression coefficients with associated *p*-values. TOS exerted a significant direct positive effect on backward reflexive saccade frequency (*β* = 1.433, *p* = 0.04), while no significant indirect effects through attention or other covariates were detected. CVDA, Composite Variable for Depression and Anxiety; D-FIS, Daily Fatigue Impact Scale.

Given the higher prevalence of vascular risk factors and mood/fatigue symptoms in the TOS group, these variables were incorporated into the models alongside age, sex, and education. The antisaccade impairment remained significant after full adjustment. The SEMs confirmed that the reduced number of correct antisaccades in TOS survivors remained significant after adjustment (*p* = 0.02; Figure 5). Attention also showed a significant direct positive influence on antisaccade performance (*p* = 0.01; Figure 5). By contrast, while TOS exerted significant direct effects on reflexive (*p* = 0.03; Figure 6) and backward reflexive saccades (*p* = 0.04; Figure 7), these outcomes were not significantly mediated by attention or other covariates. Overall, the models demonstrate that TOS status consistently impacted antisaccade and reflexive measures, whereas attentional control contributed independently only to antisaccade performance.

## Discussion

This proof-of-concept study investigated saccadic eye movements and cognitive function in TOS survivors more than four decades after exposure. The most salient finding was a selective impairment in the antisaccade task: TOS participants generated significantly fewer correct antisaccades and exhibited increased reflexive and backward reflexive saccades. In contrast, performance in visually guided and memory-guided saccades was preserved across all parameters, including latency, gain, peak velocity, and spatial error measures. These findings suggest that basic oculomotor control and spatial working memory are preserved, whereas higher-order executive functions, particularly inhibitory control, are selectively impaired. This pattern is consistent with our neuropsychological results, which identified deficits in executive function, attention, and processing speed, with memory performance remaining relatively intact. SEM models confirmed that antisaccade impairments remained significant after adjusting for confounders. Notably, attention exerted an independent positive influence only on correct antisaccade performance (Figure 5), whereas reflexive and backward reflexive saccades (Figures 6 and 7) were driven by TOS status alone. Overall, the converging oculomotor and cognitive data indicate a long-term disruption of frontal-subcortical circuits in TOS survivors.

Our findings build upon earlier TOS studies by employing objective, high-temporal-resolution eye-tracking paradigms to detect subtle executive dysfunction. They also parallel deficits observed in other immune-mediated disorders. For instance, eosinophilia–myalgia syndrome—a condition with shared toxic and immunologic mechanisms—has been associated with similar impairments in executive function and attention, alongside magnetic resonance imaging evidence of subcortical white matter abnormalities (Armstrong et al., 1997). Both syndromes likely represent a continuum of toxic-immune encephalopathies with convergent effects on prefrontal networks (Armstrong et al., 1997).

Comparable profiles have been reported in chronic exposure to organophosphate pesticides, which selectively impair attention, executive functioning, and processing speed, while sparing language and visuospatial abilities (Jamal, 1997; Ross et al., 2013; London et al., 2012). These similarities reinforce the notion that diverse environmental toxicants can lead to common neurobehavioral outcomes via frontal-subcortical disconnection. Notably, organophosphate contamination was an early suspect in TOS etiology, although it was later discredited (Gelpí et al., 2002).

The antisaccade task is a sensitive probe of frontal dysfunction, particularly implicating the dorsolateral prefrontal cortex, frontal eye fields, and basal ganglia circuits (Pierrot-Deseilligny et al., 1995). Our finding of impaired antisaccades, alongside elevated reflexive and backward saccades, indicates a failure of inhibitory control, consistent with dysfunction in frontostriatal pathways (Pierrot-Deseilligny et al., 1995). This profile echoes those seen in neurodegenerative disorders, including Parkinson’s disease dementia and dementia with Lewy bodies (Mosimann et al., 2005), Huntington disease (Patel et al., 2012) and multiple system atrophy (Brooks et al., 2017), as well as psychiatric conditions such as schizophrenia (Caldani et al., 2017; Subramaniam et al., 2018; Obyedkov et al., 2019). Similar antisaccade impairments have also been reported in prodromal or cognitive disorders, notably idiopathic REM sleep behaviour disorder (Hanuška et al., 2019) and mild cognitive impairment (Koçoğlu et al., 2021). Eye_movement abnormalities have also been noted in Alzheimer’s disease dementia (Costanzo et al., 2023) and ataxias (Szpisjak et al., 2021), underscoring the broader relevance of antisaccade deficits marker of frontal-subcortical dysfunction across diverse disorders.

While reflexive saccades are generally preserved in early Alzheimer’s disease, they tend to be impaired in subcortical and mixed dementias (Mosimann et al., 2005). The altered antisaccade pattern in TOS thus further supports selective involvement of executive networks. Functional neuroimaging and lesion studies suggest that the dorsolateral prefrontal cortex is involved in response suppression, the frontal eye fields in vector inversion, and the anterior cingulate cortex in error monitoring (Funahashi, 2006; Munoz and Everling, 2004; McDowell et al., 2008; Curtis and D’Esposito, 2003).

TOS was originally linked to aniline-contaminated rapeseed oil, triggering a systemic eosinophilic and autoimmune response with central nervous system involvement (Abaitua Borda et al., 1998; Sánchez-Porro Valadés et al., 2003; Polentinos-Castro et al., 2021; Ricoy et al., 1983; Téllez et al., 1987). Neuropathological findings described central chromatolysis in anterior horn cells and brainstem nuclei, alterations in brainstem monoaminergic centers, and non-necrotizing vasculitis with focal ischemia—findings suggestive of microangiopathy and neuroinflammation. Although consistent white matter damage was not observed, the broader pattern suggests diffuse involvement rather than localized lesions (Ricoy et al., 1983; Téllez et al., 1987).

Persistent low-grade neuroinflammation may contribute to the long-term deficits observed in TOS. Animal models of environmental neurotoxicity, including exposure to manganese and organophosphates, consistently exhibit prolonged microglial activation, astrocytosis, and elevated cytokine release (e.g., IL-6, TNF-*α*), resulting in the disruption of frontal network function (Raison et al., 2006; Zhang et al., 2023; Kirkley et al., 2017; Popichak et al., 2018; Ke et al., 2019; Sarkar et al., 2018; Conley et al., 2020; Dinamene et al., 2013). In particular, manganese neurotoxicity has been shown to induce the secretion of pro-inflammatory cytokines (TNF-α, IL-1β, IL-6) by microglia, which in turn amplifies astrocytic reactivity via NF-κB signaling and results in sustained neuroinflammation and cortical dysfunction that persists well beyond the exposure period (Kirkley et al., 2017; Popichak et al., 2018; Ke et al., 2019; Sarkar et al., 2018; Conley et al., 2020; Dinamene et al., 2013). Comparable mechanisms have been described in organophosphate models, where sustained microglia-mediated neuroinflammation amplifies neuronal hyperactivation and disrupts cortical network function long after the acute exposure (Somkhit et al., 2022). In addition, endothelial dysfunction may further compromise cerebral perfusion in metabolically demanding prefrontal regions (Iadecola, 2013).

Importantly, the absence of elevated neurodegeneration biomarkers in TOS patients argues against progressive neuronal loss, distinguishing this syndrome from Alzheimer’s disease (Ruiz-Ortiz et al., 2025). Consistent with this, preserved episodic memory and the absence of hippocampal dysfunction suggest a static or slowly progressive encephalopathy, possibly more akin to conditions such as multiple sclerosis, small vessel disease or long COVID, where frontal-subcortical networks are preferentially affected (Benito-León et al., 2025; O’Brien et al., 2003; Jolly et al., 2025; Olazarán et al., 2009). Altogether, our findings support a model of non-degenerative, immune-mediated disruption of prefrontal white matter integrity in TOS, manifesting as enduring deficits in executive function and response inhibition.

Our study’s limitations include a small sample size, which may have reduced the ability to detect subtle differences in latency or gain metrics. This limited sample size increases the risk of a Type II error and, consistent with the exploratory nature of the study, justifies not applying corrections for multiple comparisons. The cross-sectional design also restricts causal conclusions. Polyneuropathy was not treated as a confounder; although characteristic of chronic toxic oil syndrome, it is not linked to cognitive dysfunction, and adjusting for it could lead to over-adjustment and mask the true effects of the disease. Nonetheless, we acknowledge its clinical implications and report its prevalence and distribution in Table 1. Comorbidities such as hypertension, diabetes, and higher levels of anxiety, depression, and fatigue were more frequent in the TOS group. Still, these factors were incorporated into our multivariable and SEM models alongside demographic variables. Importantly, the antisaccade deficit remained significant after full adjustment, suggesting that the observed association reflects disease-related effects rather than the influence of comorbid conditions alone. However, residual confounding cannot be completely ruled out. The lack of neuroimaging data further limits structural interpretation. Future research should include both structural and functional neuroimaging to better localize and understand the observed deficits. Finally, our findings may not apply beyond the specific group of Spanish TOS survivors studied. Comparative research in other populations exposed to toxins—such as workers exposed to organophosphates or patients with eosinophilia-myalgia syndrome—could help determine whether antisaccade deficits are a common feature of chronic toxic-immune encephalopathies.

In conclusion, to our knowledge, this is the first study to apply eye-tracking to evaluate cognitive dysfunction in TOS. By combining antisaccade and related paradigms with standardized cognitive testing, we demonstrate ongoing antisaccade impairments and dysexecutive deficits consistent with disruption of frontal-subcortical control. The pattern of findings is more compatible with immune-or vascular-mediated injury than with ongoing neurodegeneration. Given that eye-tracking is objective, noninvasive, brief, and well-tolerated in older, multimorbid cohorts, antisaccade-based measures offer a practical candidate biomarker of residual executive dysfunction that now warrants validation in longitudinal, adequately powered, multimodal studies.

## Data Availability

The raw data supporting the conclusions of this article will be made available by the authors, without undue reservation.
